# Application of Machine Learning Approaches to Predict the Strength Property of Geopolymer Concrete

**DOI:** 10.3390/ma15072400

**Published:** 2022-03-24

**Authors:** Rongchuan Cao, Zheng Fang, Man Jin, Yu Shang

**Affiliations:** 1 School of Civil Engineering, Wuhan University, Wuhan 430072, China; rongchuancao@whu.edu.cn (R.C.); yushangwhu2022@163.com (Y.S.); 2 School of Civil Engineering and Architecture, Henan University, Kaifeng 475000, China; bingwu@126.com

**Keywords:** geopolymer, fly ash, concrete, modeling, machine learning

## Abstract

Geopolymer concrete (GPC) based on fly ash (FA) is being studied as a possible alternative solution with a lower environmental impact than Portland cement mixtures. However, the accuracy of the strength prediction still needs to be improved. This study was based on the investigation of various types of machine learning (ML) approaches to predict the compressive strength (C-S) of GPC. The support vector machine (SVM), multilayer perceptron (MLP), and XGBoost (XGB) techniques have been employed to check the difference between the experimental and predicted results of the C-S for the GPC. The coefficient of determination (R^2^) was used to measure how accurate the results were, which usually ranged from 0 to 1. The results show that the XGB was a more accurate model, indicating an R^2^ value of 0.98, as opposed to SVM (0.91) and MLP (0.88). The statistical checks and k-fold cross-validation (CV) also confirm the high precision level of the XGB model. The lesser values of the errors for the XGB approach, such as mean absolute error (MAE), mean square error (MSE), and root mean square error (RMSE), were noted as 1.49 MPa, 3.16 MPa, and 1.78 MPa, respectively. These lesser values of the errors also indicate the high precision of the XGB model. Moreover, the sensitivity analysis was also conducted to evaluate the parameter’s contribution towards the anticipation of C-S of GPC. The use of ML techniques for the prediction of material properties will not only reduce the effort of experimental work in the laboratory but also minimize the cast and time for the researchers.

## 1. Introduction

Geopolymer is generally considered the third generation of cement, following gypsum cement and regular Portland cement (OPC) [1,2,3]. It has emerged as a significant construction material on a global scale [4,5,6,7]. Geopolymers are also known as amorphous alkali aluminosilicates and alkali-activated cement [8,9,10,11,12,13]. By activating aluminosilicates, such as rice husk ash (RHA), metakaolin (MK), slag (SG), and fly ash (FA) with an alkaline solution, geopolymer concrete can be created. As a result, the effectiveness of GPC production normally relies on activators and the type of aluminosilicates available [14,15,16,17,18,19].

Geopolymer is a type of inorganic polymer. In comparison to other natural zeolitic materials, it is amorphous rather than crystalline [20,21,22,23,24,25,26]. Polymerization involves a very rapid reaction between silica (Si) and alumina (Al) in an alkaline environment, which results in the formation of a three-dimensional polymeric chain of Si-O-Al-O links. In contrast to OPC or pozzolanic cement, geopolymer achieves compressive strength by the polycondensation of alumina, silica, and a high alkali concentration [27,28,29,30]. By contrast, a geopolymer including OPC forms calcium silicate hydrates (C-S-H), polycondensation of silica and alumina, and a high alkali content to achieve compressive strength. GPC can be prepared from anything which has amorphous Al and Si. Minerals obtained from natural resources or by-products from industries can be used as these materials. The hydration products of FA/MK were discovered to be sodium aluminosilicate hydrate gels. Meanwhile, calcium silicate gels of hydrate are the products of hydration for SG activation [8].

MK-based GP is superior to hydrates due to its more lasting characteristics [7,31,32,33,34,35]. Despite its advantages, it resulted in serious rheological issues due to the increased water demand. Meanwhile, geopolymers based on FA demonstrated increased durability [7,28,36,37,38]. On the other side, a polymer-based on SG has stronger initial strength and acid resistance [7,39,40,41,42]. Numerous research has been conducted to evaluate the geopolymer concrete performance. These impacts include those of the C-S-H range, chemicals, and type of curing. Yip et al. [23] discovered aluminosilicate gel (N-A-S-H) and C-S-H in MK/SG-based geopolymer pastes. This is comparable to a rich calcium FA-based geopolymer reported by Somna et al. [43], which is activated primarily with sodium hydroxide (NaOH). The N-A-S-H and C-S-H components of concrete paste contribute to its strength. In other words, GP pastes’ strength strongly relies on the alkalinity of the utilized activators. Additionally, it was also noted that temperature plays a vital role in the effect on the activation of aluminosilicates. At a temperature of about 27 °C (lower), the activation process in FA/SG blends is in control of SG activation, but at the temperature of about 60 °C (high), both SG and FA are activated.

**Figure 1:**
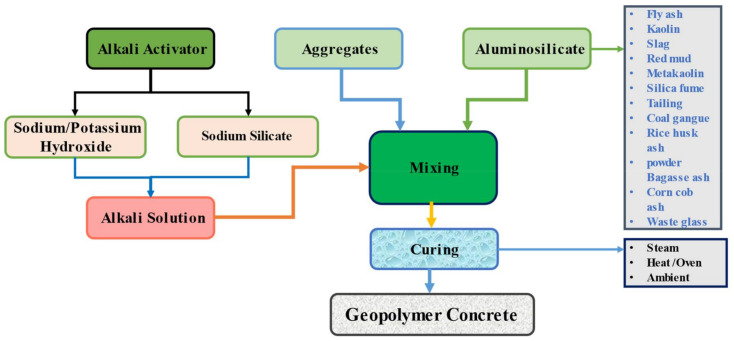
Manufacturing procedure of the geopolymer concrete (Wang, Q., et al. 2022).

SG, on the other hand, contributes to the strength of pastes due to its compact microstructure [44,45,46]. The formation of C-S-H and C-A-S-H causes the solidification of FA/SG-based GP. C-S-H, N-A-S-H, and C-A-S-H are created after hardening. Furthermore, the formation of hydration gels is influenced by calcium ions and pH. According to Prinya et al. [47], in FA-based GP, an acidic environment causes the development of N-A-S-H gel. Calcium ion concentrations are higher in class geopolymers that contain C. Increased C-S may occur as a result of FA [48]. The higher potassium oxide concentration of H.CWA aided the strength growth [49] and self-activation of the geopolymer with no need for an activator [50]. Additionally, the use of secondary cementitious materials and fibers (both natural and artificial) in geopolymer concrete has shown effective performance [51,52,53,54,55,56]. However, Figure 1 shows a schematic illustration of the GP concrete manufacturing process.

In the meantime, the rapid surge towards the use of various ML techniques for the prediction of numerous properties of materials plays a vital role for researchers in the field of engineering [57,58,59,60,61,62,63,64]. Especially the prediction of mechanical properties of different types of concrete, as concrete is a material that requires experimental efforts, time, and cost to achieve the desired strength [7,60,62,65,66,67,68,69,70,71]. The various types of software and codding are being developed to generate the different types of ML algorithms, such as AdaBoost, bagging, decision tree, MLP, GEP, and ANN. Bayar et al. [72] predicted the various crack propagation of concrete material and investigated that the use of employed ML approaches predicted the crack propagation effectively. Seung et al. [73] used the ANN approach for the anticipation of the concrete’s strength. The type of pattern system was generated for ANN, which can learn the cylinder test results. They demonstrate that I-Pre-Cons (Intelligent Prediction system of Concrete Strength), when combined with ANN, is extremely effective at predicting the C-S growth of concrete. Nguyen-Sy et al. [74] used the extreme gradient boosting approach for predicting the C-S of concrete. They explained that the XGB approach is more resilient and has higher precision than the ANN and SVM techniques and other machine learning methods currently available in the literature.

This research describes the comparative study of various ML algorithms for predicting the C-S of GPC. The objectives of the study are as follows:

- To investigate the combined effect of ensemble and individual ML algorithms for predicting the strength property of concrete.
- To evaluate the more precious ML algorithm for predicting the compressive strength of concrete.
- To minimize the experimental efforts, cost, and time with the application of employed ML approaches.

## 2. Research Strategy

**Figure 2:**
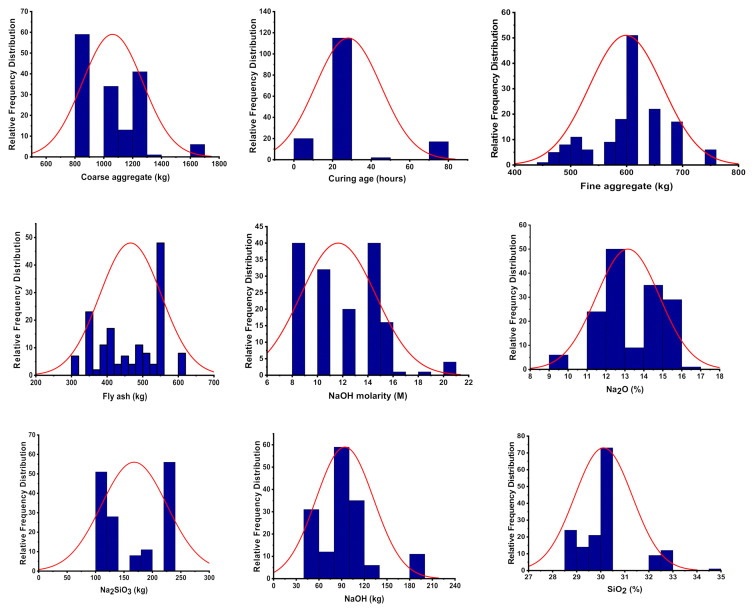
Input parameter’s relative frequency distribution.

The concrete material was prepared and used in the study with the nine parameters (coarse aggregate, FA, fine aggregate, sodium hydroxide, Na_2_SiO_3_, silicon dioxide, Na_2_O, NaOH molarity, and curing time) to obtain the C-S, as described in the literature [75,76,77,78,79,80,81,82,83,84]. A total of 151 data points has been collected from the mentioned literature for running the selected models. The said retrieved data were arranged as per the requirement of the anaconda navigator software. The spyder (4.1.4) from the same software was investigated for running the models using python codding for each model. The nine columns were arranged as input parameters, while a single column was used as output (C-S) variable in an excel sheet for modeling purposes. The XGBoost, SVM, and MLP algorithms were used for the prediction of required output (C-S). The predicted result of C-S was then obtained in the form of an R^2^ value, which normally varies from 0 to 1. The higher value of R^2^ is the reflection of better results with high precision of the selected model [85,86,87]. Table 1 contains a descriptive statistical analysis of all input parameters. All attributes are expressed in kilograms, except for the age in days, which is specified in the table. Moreover, the histograms in Figure 2 indicate the relative frequency distribution of each variable.

## 3. Machine Learning Algorithms

The two different types of ML approaches were selected for this study; one is individual type (MLP), while another is ensemble ML techniques (XGBoost and SVM) to evaluate and recommend the better model for the prediction of the required outcome.

### 3.1. Multilayer Perceptron Regressor (MLP)

MLPs are normally the feedforward neural network. Networks containing numerous layers of a perceptron are known as multilayer perceptrons (MLPs) (with threshold activation). Vanilla neural networks are multiple perceptrons with only one hidden layer. An MLP has three levels of nodes: input, hiding, and outputs. Each node, with the exception of the input nodes, is a neuron with a nonlinear activation function. Backpropagation is an MLP supervised learning approach. MLPs have more layers and nonlinear activation than linear perceptrons. It can split data that are not linearly separable. If each neuron in an MLP has a linear type activation function, then linear algebra confirms that the number of layers may be minimized to a two-layer input and output model. MLP neurons use a non-aligned activation function to replicate action potentials or firing frequency of genuine neurons.

### 3.2. Support Vector Machine (SVM)

SVMs are controlled learning models that analyze data for allocation and regression. SVMs are among the most popular and resilient anticipation approaches since they are founded on statistical learning frameworks. The SVM technique for training generates a type of model that allows fresh specify to one of two categories, transforming it to a non-probabilistic binary linear classifier. SVM assigns training instances to places in space to maximize the distance between two groups. Then new examples are summarized into the same space and categorized according to their gap. SVMs may also conduct the fast nonlinear type of classification employing the kernel trick, which involves essentially mapping inputs into high-dimensional feature spaces. Unlabeled data make supervised learning unfeasible hence an unsupervised learning technique is necessary to uncover natural clustering and then by mapping fresh data for these groups. The SV clustering algorithm uses the same statistics as the support vector machines approach to identify unlabeled data. 

### 3.3. XGBoost Algorithm

XGBoost is an ensemble ML approach and is based on DTs and used a GB framework. ANNs normally outperform all other techniques or frameworks in anticipating problems, including non-structured data (pictures, text, etc.). For minimum-to-normal-sized tabular data, DT-based algorithms are now rated best-in-class. XGB parallelizes the action of sequential tree construction because the loops employed to produce base learners are compatible; the outer loop specifies the leaf nodes of a selected tree; however, another loop (inner) investigates the features. This process of loops prohibits correlations because the loop (outer) cannot be taken to start until the inner loop is completed (the more arithmetically expensive of the two). Due to this, the pattern of loops is regulated to enhance the run time by initialization through a global scan of all occurrences and sorting via aligned threads. This trend increases algorithmic speed by remunerating for any parallelization overheads elaborated during the investigation. This algorithm was developed in order to produce the best possible use of the resources related to hardware that was available. This is performed via cache awareness, with each thread assigning internal buffers for holding gradient statistics. Additional innovations, such as ‘out-of-core’ computation, maximize available disc space while managing large data-frames that are too large to fit in memory.

## 4. Result and Discussions

### 4.1. MLP Model Outcome

**Figure 3:**
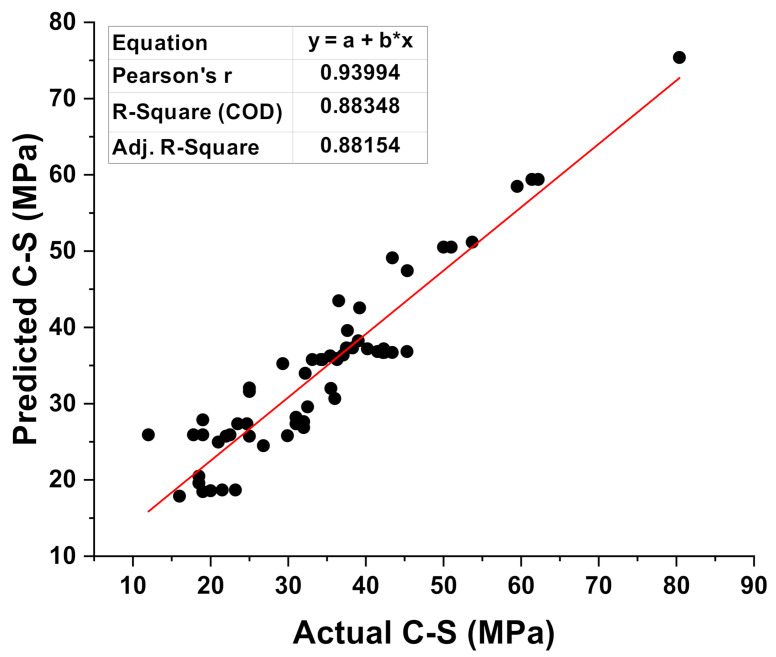
Correspondence between the experimental and predicted result of the MLP model.

**Figure 4:**
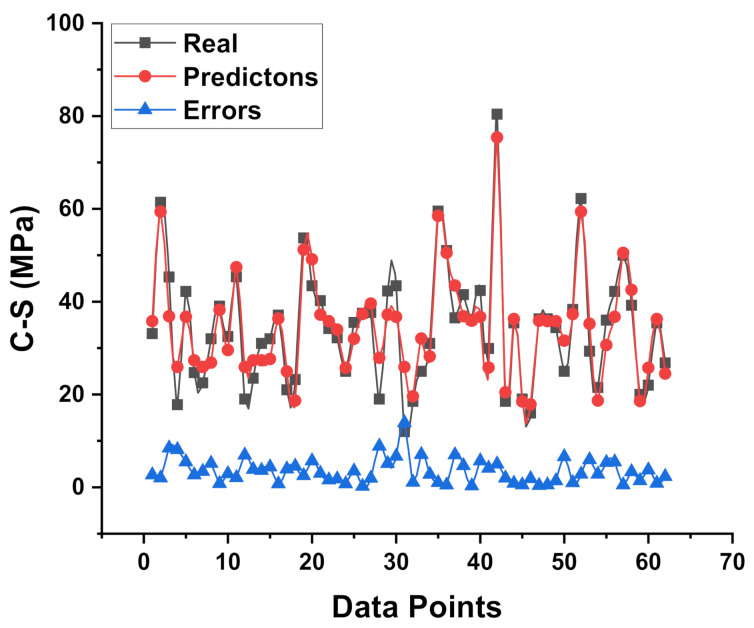
Experimental and predicted dispersal of the results along with the scattering of their errors for the MLP model.

Figure 3 depicts the analysis explanation of the actual and predicted findings for the C-S of GPC for the MLP model. The MLP model gives outcomes with a reasonable degree of correctness and a minor difference between the actual and projected values. The R^2^ score of 0.88 suggests that the MLP model’s result is at reasonably high accuracy for predicting outcomes. Figure 4 shows the dispersion of the values obtained from the experimental approach (targets), anticipated results, and error results for the model (MLP). The high, minimum, and average values of the error’s scattering for the dataset were reported as 13.91 MPa, 0.19 MPa, and 3.48 MPa, respectively. However, it was also noted that the set shows 20.96% of the data between 0 to 1 MPa, 30.64% between 1 MPa and 3 MPa, while 48.38 percent the data was above 3 MPa.

### 4.2. SVM Model Outcome

**Figure 5:**
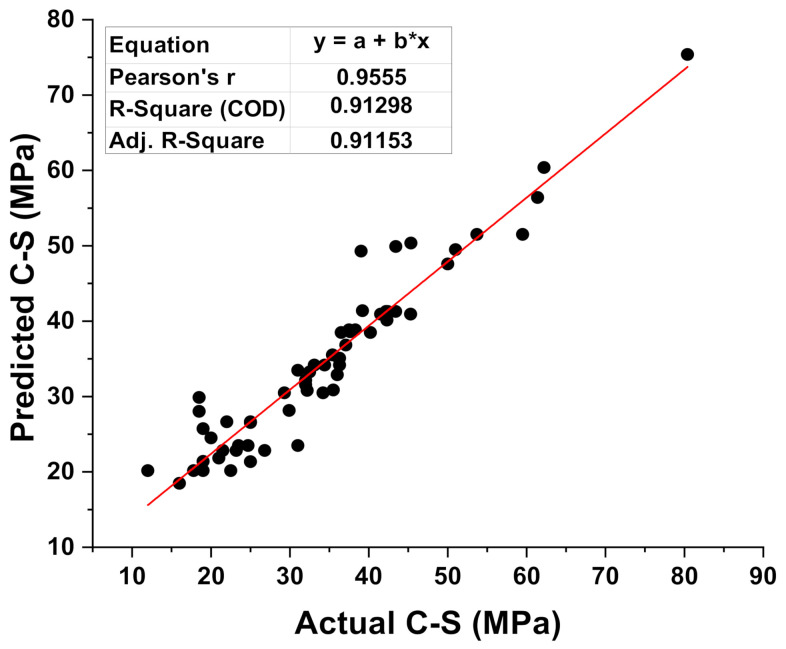
Correspondence among the experimental and predicted results of SVM model.

**Figure 6:**
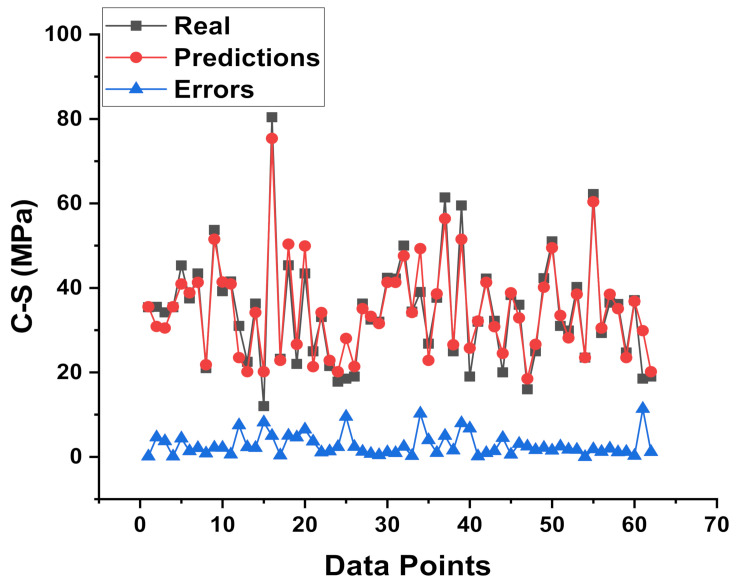
Experimental and predicted dispersal of the results along with the scattering of their errors for the SVM model.

Figure 5 and Figure 6 compare the actual and expected outputs of the SVM model. Figure 5 gives the information of the correlation between results (experimental) and predictions from the SVM model, indicating the R^2^ value of 0.91. This result of SVM is the reflection of more accuracy as opposed to MLP model output. The spread in the form of colored dots of experimental values (real), predicted outcomes from the SVM model, and difference values between these are depicted in Figure 6. In addition, the lower, maximum, and average values of the errors for the set were noted as 0.025 MPa, 5.0 MPa, and 1.49 MPa, respectively. Moreover, 32.2% of the data was lying between 0 and 1 MPa, 59.67% data was reported among the 1 MPa and 3 MPa, while only 8.064% data was lying above 3 MPa.

### 4.3. XGBoost Model Outcome

**Figure 7:**
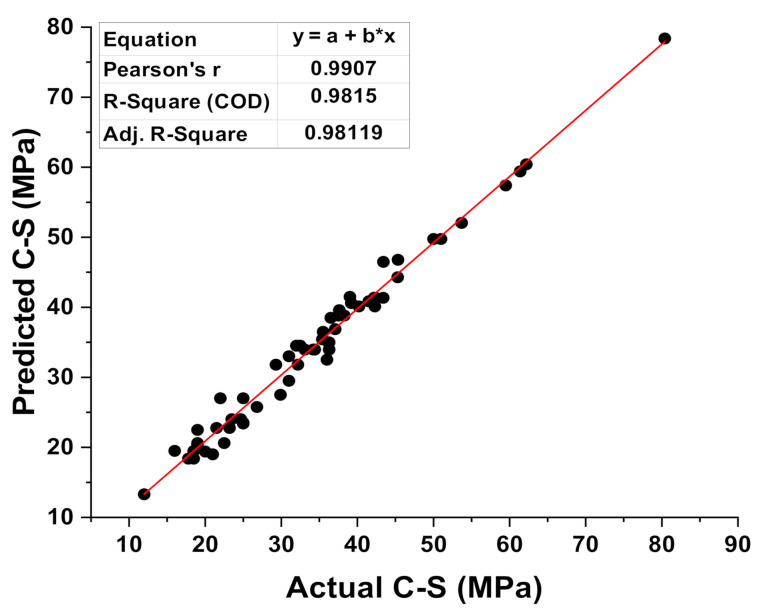
Correspondence among the experimental and predicted result of XGB model.

**Figure 8:**
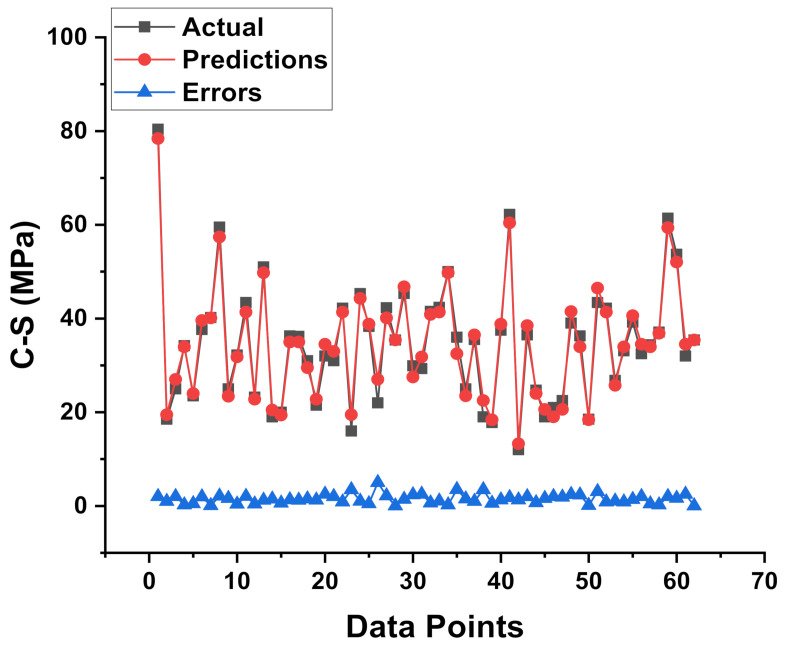
Experimental and predicted dispersal of the results along with the scattering of their errors for the XGB model.

Figure 7 illustrated the connection between the results of C-S from the various mixes through experimental work and anticipated C-S results obtained from the XGB model (predicted). This model gives an R^2^ value of 0.98, showing that it was more accurate at predicting outcomes than the MLP and SVM models. The dispersal of the colored dots for the results obtained from the various mixes during the experimental approach results generated from the XGB model (predicted), and the difference of these two results is shown in Figure 8. It was also reported that the distribution of the errors gives the maximum, lowest, and average results for the selected set as 11.37 MPa, 0.005 MPa, and 2.77 MPa, respectively. In the meantime, 24.19% of the data was noted between 0 and 1 MPa, 45.16% of data was lying between 1 MPa and 3 MPa, and 30.64% of the data was noted above 3 MPa.

## 5. K-Fold Cross-Validation (CV) Approach

K-fold CV is a widely used strategy among data scientists. It is a data partitioning approach that enables the efficient use of datasets in order to construct a more extended model. The primary goal of any type of machine learning is to create a more generic model capable of performing well on unknown data. While it is possible to develop a flawless model with 100 percent accuracy or zero error on training data, it may fail to generalize unobserved data. As a result, it is an inadequate model. It produces overfitting of the training data. Machine learning is all about generalization, which means that the performance of a model may be determined only using data points that were not utilized during the training process. Therefore, the data are frequently split into a training and a test set. With k-fold cross-validation, the data splitting procedure may be performed more effectively.

The dataset is subdivided into k subsets, and the holdout approach was used for each subset k times. At every stage of time, single k subsets were used as the test set, while the other k-1 subsets comprised the training. The error average for all trails (k) is then evaluated. The importance of this trend is that it is less important than how the data are separated. Data points normally show his appearance only one time in a test set, while it shows k-1 time his appearance in the training set. The increase in the k showed a decrease in the variance of the resulting estimate. The drawback of this process is that the training algorithm should run k times from scratch, which implies that doing an evaluation requires k times as much work. A variation on this strategy is to split the data randomly into a test and training set k times. The advantage of this approach is that you can separately determine the size of each test set and the number of trials to average. In addition, the statistical measure for the ML approaches is illustrated in Table 2.

Statistical analysis was used to evaluate the models’ predictive ability in co-occurrence with the following Equations (1)–(3).

(1) $$\mathrm{RMSE}\;=\;\sqrt{\frac{\sum_{i\;=\;1}^{n}\;{\left(ex_{i}\;-\;mo_{i}\right)}^{2}}{n}}$$

(2) $$\mathrm{MAE}\;=\;\frac{\sum_{i\;=\;1}^{n}\left|ex_{i}\right.-\left.mo_{i}\right|}{n}$$

(3) $$R\;=\;\frac{\sum_{i\;=\;1}^{n}\left(ex_{i}-{\bar{ex}}_{i}\right)\left(mo_{i}-{\bar{mo}}_{i}\right)}{\sqrt{\sum_{i\;=\;1}^{n}{\left(ex_{i}-{\bar{ex}}_{i}\right)}^{2}\sum_{i\;=\;1}^{n}{\left(mo_{i}\;-\;{\bar{mo}}_{i}\right)}^{2}}}$$

where, $ex_{i}$, $mo_{i}$, ${\bar{ex}}_{i},\;{\bar{mo}}_{i},\;$ and n are the actual, predicted, mean actual, mean predicted values, and the number of samples, respectively.

**Figure 9:**
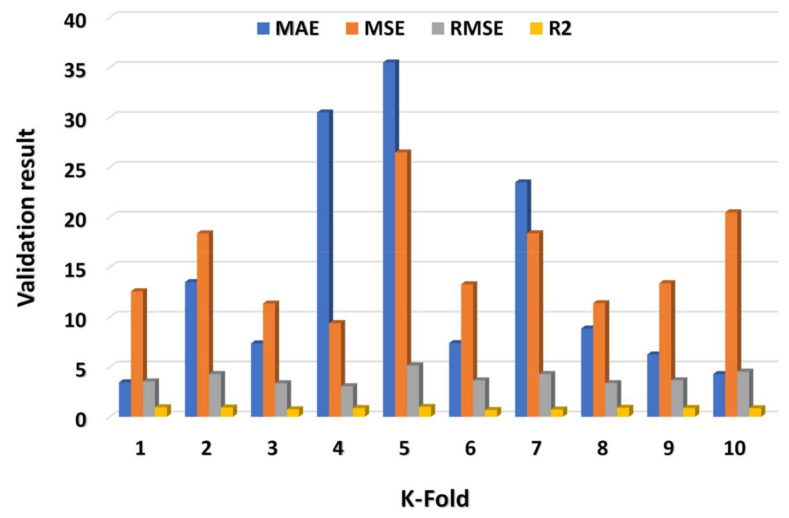
MLP model’s statistical explanation of CV analysis.

**Figure 10:**
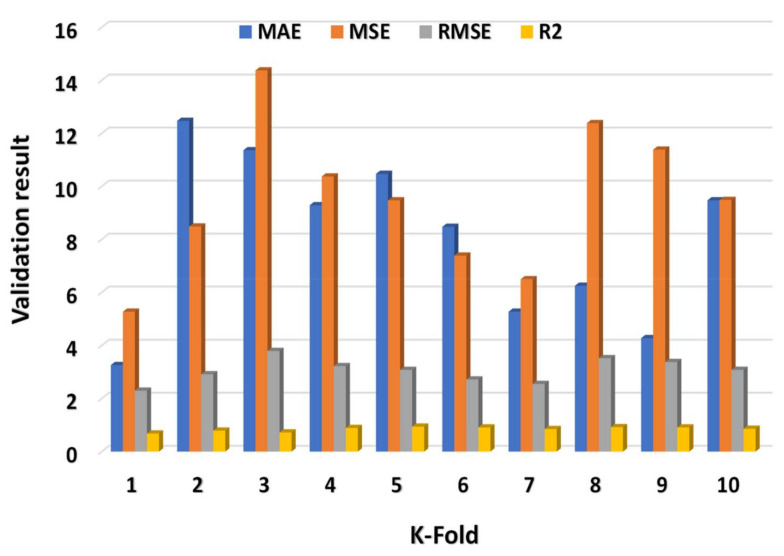
SVM model’s statistical explanation of CV analysis.

**Figure 11:**
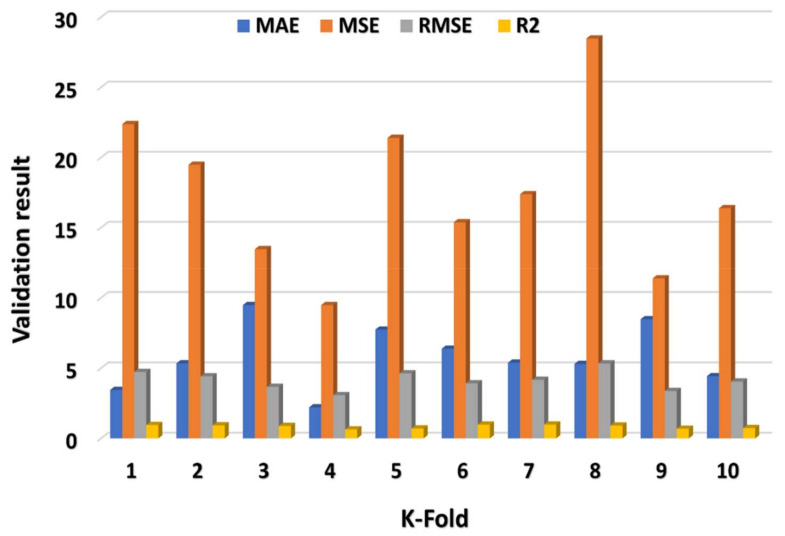
XGB model’s statistical explanation of CV analysis.

The MAE, R^2^, MSE, and RMSE values were utilized to assess CV, and their dispersal for MLP, SVM, and XGB model’s outcome are shown in Figure 9, Figure 10 and Figure 11, respectively. The XGB algorithm showed the minimum error and came up with a high R^2^ value, indicating it is the most effective predictive approach. As illustrated in Figure 11, the highest, minimum, and average R^2^ values for the MLP model were 0.99, 0.68, and 0.86, respectively. The highest, lower, and average R^2^ results for the SVM model were 0.94, 0.68, and 0.85, respectively, as shown in Figure 9. In contrast, the XGB model’s greatest, lowest, and average R^2^ values were 0.99, 0.64, and 0.84, respectively, as illustrated in Figure 11. Additionally, the MAE, MSE, and RMSE maximum values for the MLP model were 35.48 MPa, 26.48 MPa, and 5.15 MPa, respectively (Figure 9), whereas the SVM model’s MAE, MSE, and RMSE maximum values were 12.47 MPa, 14.37 MPa, and 3.79 MPa, respectively (Figure 10). The MAE, MSE, and RMSE values for the XGB model, on the other hand, were 9.48 MPa, 28.48 MPa, and 5.34 MPa, respectively. This also validated the XGB model’s high precision in predicting the C-S of GPC.

## 6. Sensitivity Analysis

**Figure 12:**
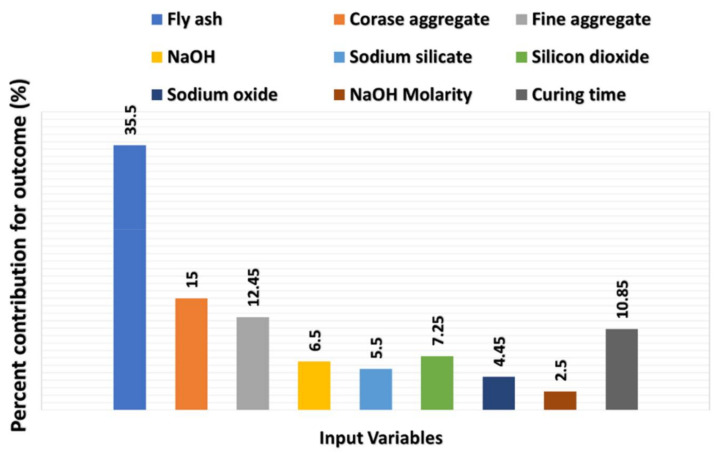
Contribution level in percentage towards the predicted outcome.

The purpose of this analysis is to ascertain the effect of input parameters on GPC C-S anticipating. The variables (input) have a substantial influence on the predicted results [62]. The impact of each parameter on the C-S predicting is shown in Figure 12. The results indicated that FA contributed the most (35.5 percent), followed by coarse aggregate (15 percent) and fine aggregate (12.45 percent). The remaining variables, on the other hand, come up with significantly less to the prediction of GPC C-S, with molarity accounting for 2.5 percent, Na_2_SiO_3_ accounting for 5.5 percent, curing time accounting for 10.85 percent, NaOH accounting for 6.5 percent, SiO_2_ accounting for 7.25 percent, and Na_2_O accounting for 4.45 percent. Sensitivity analyses produce results that are proportional to the input variables and total dataset employed to construct the model. Nevertheless, the ML algorithm detects the influence of every setting. With the variation in the proportion of mixes and the inclusion of new parameters, these analyses generate inconsistent results. The participation of variables in the model’s results was calculated using the following Equations (4) and (5).

(4) $$N_{i}\;=\;f_{max}\left(x_{i}\right)\;-\;f_{min}\left(x_{i}\right)$$

(5) $$S_{i}\;=\;\frac{N_{i}}{\sum_{j\;-\;i}^{n}N_{j}}$$

## 7. Discussion

The motive of this research was to demonstrate the utility of both individual and ensemble ML algorithms for estimating the C-S of GPC, a geopolymer binder that was designed to be used in the concrete manufacturing process in place of cement. The objective was to create a type of material that is environmentally friendly and cement-free. This work predicted the C-S of GPC with MLP, SVM, and XGB ML algorithms. The number of input parameters has a significant effect on the required outcome as reported in the literature [88]. The number of input variables can be enhanced by incorporating the other environmental effects, such as temperature and humidity. The XGB model’s output showed high accuracy, with an R^2^ value of 0.98, opposed to 0.91 for the SVM technique and 0.88 for the MLP approach. The high accuracy of the XGB model has also been reported in the literature [89,90]. In contrast, the performance was examined of the MLP, SVM, and XGB models using analysis (statistical) and the k-fold CV technique. The minimum result of the errors (RMSE, MAE, MSE) is also the confirmation of a more accurate model. However, evaluating and recommending the ideal ML regressor for anticipating results across a variety of fields is challenging, as the parameters and dataset play a vital role in the model’s accuracy. Moreover, ensemble ML algorithms frequently make use of the weak learner by producing sub-models for training on data and optimizing for the highest R^2^ value. Additionally, research depicts that the XGB technique shows higher accuracy than other ML techniques. Moreover, additional analysis (sensitivity) was done to investigate the effect of parameters on the projected C-S of GPC. The result of the selected models may be influenced by the model parameters and selected dataset. This analysis identifies which of the nine parameters (input) has the greatest influence on the predicted output. The python coding can also be arranged to evaluate or predict any type of output based on the provided input parameters.

## 8. Conclusions

This research describes the application of different ML approaches on the data of GP concrete retrieved from the literature. A total of 3 types of ML techniques were investigated on the 146 data points. The MLP, SVM, and XGB ML algorithms were used on the same data to predict the C-S of GP concrete. The below-mentioned conclusions can be drawn from the research:

- The XGB model performs effectively and preciously towards the prediction of C-S of GP concrete;
- The R2 result of XGB equals 0.98 and is a reflection of its high-level performance as opposed to the R2 value of SVM (0.92) and MLP (0.88);
- The statistical analysis and K-fold CV approach also confirm the accurate prediction of the XGB model;
- The lower values of the statistical results in the form of errors, such as MAE, MSE, and RMSE, also give a reflection of the high precision of the XGB model for anticipating the C-S of GP concrete;
- The sensitivity analysis shows that the maximum contributing input parameter was fly ash crossing 35% towards the prediction of C-S of GP concrete.

Moreover, the recommended ML approach is the XGB, which shows effective results towards the anticipation of C-S of concrete. The precision level of the selected algorithms can also be enhanced by increasing the dataset and input parameters. The other statistical checks, such as singular spectrum analysis (SPA), with the inclusion of other statistical metrics, such as normalized root-mean-square error (NRMSE), coefficient of variation (COV), overall index (OI), efficiency coefficient (EC), mean relative error (MRE), and residual mass coefficient (RMC), can also be applied to cross-verify the obtained results from the selected models [91]. The other ML approaches, such as ANN, Adaboost, and bagging regressor, can also be investigated to check the accuracy level for the prediction of required outcomes. 

## Figures and Tables

**Table 1:** Descriptive statistics of the input variables.

Parameters	Fly Ash	CA	FA	NaOH	Na_2_SiO_3_	SiO_2_	Na_2_O	Molarity	CT
Mean of variables	465.79	1060.99	598.93	94.26	167.87	30.12	13.16	11.65	28.13
Variable’s median	494.00	1091.00	600.00	95.00	138.00	30.00	12.00	12.00	24.00
Variable’s mode	550.00	838.00	600.00	95.00	239.00	30.00	12.00	8.00	24.00
Standard Deviation	86.54	210.13	65.61	38.05	57.61	1.20	1.67	2.98	17.01
Standard Error	6.97	16.93	5.29	3.07	4.64	0.10	0.13	0.24	1.37
Input ranges	300.00	846.00	291.00	157.00	136.00	6.00	7.20	12.00	69.00
Lowest values	300.00	838.00	459.00	41.00	103.00	28.70	9.00	8.00	3.00
Highest values	600.00	1684.00	750.00	198.00	239.00	34.70	16.20	20.00	72.00

**Table 2:** Statistical measures of the employed models.

ML Approaches	MAE (MPa)	MSE (MPa)	RMSE (MPa)
MLP	3.48	19.0969	4.37
XGB	1.49	3.1684	1.78
SVM	2.77	14.5924	3.82

## Data Availability

Not applicable.
